# Can roflumilast, a phosphodiesterase-4 inhibitor, improve clinical outcomes in patients with moderate-to-severe chronic obstructive pulmonary disease? A meta-analysis

**DOI:** 10.1186/s12931-016-0330-y

**Published:** 2016-02-17

**Authors:** Jian Luo, Ke Wang, Dan Liu, Bin-Miao Liang, Chun-Tao Liu

**Affiliations:** Department of Respiratory and Critical Care Medicine, Sichuan University, Chengdu, China; Department of Critical Care Medicine, West China School of Medicine and West China Hospital, Sichuan University, Chengdu, China; No. 37 Guoxue Alley, Chengdu, 610041 China

**Keywords:** Roflumilast, Chronic obstructive pulmonary disease, Lung function, Exacerbation, Meta-analysis

## Abstract

**Background:**

Effects of roflumilast on lung function, symptoms, acute exacerbation and adverse events in patients with chronic obstructive pulmonary disease (COPD) are controversial. We aimed to further clarify the efficacy and safety of roflumilast in treatment of moderate-to-severe COPD.

**Methods:**

From 1946 to November 2015, we searched the Pubmed, Embase, Medline, Cochrane Central Register of Controlled Trials, ISI Web of Science and American College of Physician using “roflumilast” and “chronic obstructive pulmonary disease” or “COPD”. Randomized controlled trials that reported forced expiratory volume in one second (FEV_1_), forced vital capacity (FVC), transition dyspnea index (TDI), St George’s Respiratory Questionnaire (SGRQ), and incidence of COPD exacerbations and adverse events were eligible. We conducted the heterogeneities test and sensitivity analysis, and random-effects or fixed-effects model was applied to calculate risk ratio (RR) and mean difference (MD) for dichotomous and continuous data respectively. Cochrane systematic review software, Review Manager (RevMan), was used to test the hypothesis by Mann-Whitney *U*-test.

**Results:**

Thirteen trials with a total of 14,563 patients were pooled in our final studies. Except for SGRQ (*I*^2^ = 63 %, *χ*^2^ = 1.71, *P* = 0.07) and adverse events (*I*^2^ = 94 %, *χ*^2^ = 0.03, *P* < 0.001), we did not find statistical heterogeneity in outcome measures. The pooled MD of pre- and post-bronchodilator FEV_1_ was 54.60 (95 % confidence interval (CI) 46.02 ~ 63.18) and 57.86 (95 % CI 49.80 ~ 65.91), and both showed significant improvement in patients with roflumilast (*z* = 12.47, *P* <0.001; *z* = 14.07, *P* < 0.001), so did in FVC (MD 90.37, 95 % CI 73.95 ~ 106.78, *z* = 10.79, *P* < 0.001). Significant alleviation of TDI (MD 0.30, 95 % CI 0.14 ~ 0.46, *z* = 3.67, *P* < 0.001) and decrease of acute exacerbation (RR 0.86, 95 % CI 0.81 ~ 0.91, *z* = 5.54, *P* < 0.001) were also identified in treatment of roflumilast, but without significant difference in SGRQ (MD −1.30, 95 % CI −3.16 ~ 0.56, *z* = 1.37, *P* = 0.17). Moreover, roflumilast significantly increased the incidence of adverse events compared with placebo (RR 1.31, 95 % CI 1.16 ~ 1.47, *z* = 4.32, *P* < 0.001).

**Conclusions:**

Roflumilast can be considered as an alternative therapy in selective patients with moderate-to-severe COPD.

## Background

Chronic obstructive pulmonary disease (COPD), a common disease with a prevalence reported to be 7.8 to 19.7 %, is characterized by persistent and progressive airflow limitation as well as frequent exacerbations [1, 2]. Global Burden of Disease (GBD) Study projected that COPD will become the third leading cause of death worldwide by 2020, and it was estimated as the direct underlying cause of 7.8 % of all deaths and 27 % of deaths related with smoking [3, 4].

Acute exacerbation of COPD is defined as respiratory symptoms deterioration and medication alteration, and it has been demonstrated to be associated with detriment of quality of life, decline of lung function and increase of mortality [1, 5–7]. Hurst and his colleagues analyzed 2138 patients with COPD, and they found a trend of more exacerbations as the severity of COPD increased, that was 22, 33 and 47 % in stage 2 (moderate), stage 3 (severe) and stage 4 (very severe), respectively [8]. Therefore, effective treatment and management in patients with moderate-to-severe COPD is paramount to decrease exacerbations, and improve lung function, quality of life and clinical outcomes.

Phosphodiesterase-4 (PDE4) is a vital enzyme in the metabolism of cyclic adenosine monophosphate (cAMP) and inhibition of PDE4 can inactivate immune and inflammatory cells via increase cAMP [9]. It is recommended by the Global Initiative for Chronic Obstructive Lung Disease (GLOD) guideline that a combination of PDE4 inhibitor and long-acting bronchodilator can be considered as an alternative treatment in patients with severe COPD due to the effective improvement of lung functions [1]. Roflumilast is a novel selective inhibitor of PDE4, which functions mainly by its active metabolite, roflumilast N-oxide, via the conversion by cytochrome P450 (CYP) 3A4 and 1A2 isozymes [10]. Rabe and his colleagues conducted a randomized controlled trial (RCT) in 1157 patients with moderate-to-severe COPD, and they found that roflumilast could significantly improve post-bronchodilator forced expiratory volume in one second (FEV_1_) (0.097 ± 0.018, *P* < 0.0001) and post-bronchodilator forced vital capacity (FVC) (0.114 ± 0.031, *P* = 0.0002), and decrease incidence of acute exacerbations (28 % vs. 35 %, *P* = 0.0114) compared with placebo [11], which were further demonstrated by a subsequent meta-analysis of seven trials with 9675 patients but without improving health-related quality of life by St George’s Respiratory Questionnaire (SGRQ) (mean difference (MD) −0.70, 95 % confidence interval (CI) −2.65 ~ 1.26, *P* = 0.49) or decreasing mortality rate (risk ratio (RR) 0.90, 95 % CI 0.63 ~ 1.29, *P* = 0.56) [12].

However, Fabbri and his colleagues randomly assigned 743 patients with moderate-to-severe COPD into roflumilast plus tiotropium and tiotropium groups and they reported a significant improvement in Shortness of Breath Questionnaire (SOBQ) (MD −2.6, 95 % CI −4.5 ~ −0.8, *P* = 0.0051) in roflumilast plus tiotropium [13]. Moreover, a recent placebo-controlled randomized study, which investigated the additional treatment of roflumilast in moderate-to-severe COPD with chronic bronchitis, did not reveal any significant changes in lung function, quality of life, or exercise tolerance between rolumilast and placebo [14]. Therefore, the accurate roles of roflumilast in the treatment of patients with COPD still remain controversial.

In this study, we conducted a meta-analysis of all published RCTs with the aim of updating and further clarifying the efficacy and safety of roflumilast in patients with COPD.

## Methods

Our study protocol was approved by the Institutional Ethical Committee for Clinical and Biomedical Research of West China Hospital (Sichuan, China), so did in each enrolled trial by the corresponding institutional review board. All participants provided written informed consent.

### Search strategies

From 1946 to November 2015, a comprehensive computer search was conducted in Pubmed, Embase, Medline, Cochrane Central Register of Controlled Trials (CENTRAL), ISI Web of Science and American College of Physician (ACP) using the keywords of “roflumilast” and “chronic obstructive pulmonary disease” or “COPD” with limitation in the publication type of RCTs but not in the publication language. We reviewed the references listed in each identified article and manually searched the related articles to identify all eligible studies and minimize the potential publication bias.

### Inclusion and exclusion criteria

Eligible clinical trials were defined based on the following criteria: 1) study design was RCT; 2) moderate-to-severe COPD was diagnosed by physicians according to the guidelines released by GOLD with a post-bronchodilator FEV_1_ between 30 and 80 % [1]; 3) age was more than 40 years old and smoking history was more than 10 pack-years; and 4) intervention treatment was oral roflumilast with a dose of 500ug and a frequency of once daily, but regardless of administration durations. We did not enroll trials that were retrospective, observational, cohort or case control studies.

### Outcome measures

Outcome measures consisted of efficacy assessment and safety evaluation, which included: 1) change of lung functions from baseline, such as pre-bronchodilator FEV_1_ and post-bronchodilator FEV_1_, FVC, force expiratory volume in six seconds (FEV_6_) and forced expiratory flow between 25 and 75 % of the vital capacity (FEF_25-75_); 2) health-related quality of life such as investigator-administered transition dyspnea index (TDI) and SGRQ, and 3) incidence of COPD exacerbations and adverse events.

### Study selection

Two independent investigators performed the study selection in two phases. Firstly, they discarded duplicated and non-randomized controlled studies by screening titles and abstracts. Secondly, eligible studies were extracted by reviewing full texts in accordance with the previously designed study inclusion criteria. Any disagreement was solved by mutual consensus in the presence of a third investigator.

### Data extraction

Independently, two data collectors extracted and recorded desirable information of each enrolled study in a standard form recommended by Cochrane [15], which consisted of authors, publication year, registration series, study design, participants and population, demographic characteristics (age, gender, etc.), baseline characteristics (FEV_1_/FVC, post-bronchodilator FEV_1_, post-bronchodilator FEV, etc.), details of intervention treatment (dose, frequency, routine, and duration), follow-up period, and outcome measures and study results. For any missing data information, corresponding authors were contacted by email to request the full original data. Different opinions between the two collectors were determined by reaching a consensus or consulting a third investigator.

### Quality assessment

For the assessment of risk of bias in estimating the study outcomes, we used the Cochrane risk of bias tool [15]. Each study was assessed for: 1) random sequence generation (selection bias); 2) allocation concealment (selection bias); 3) blinding of participants and personnel (performance bias); 4) blinding of related outcomes assessment (detection bias); 5) incomplete outcome data (attrition bias); 6) selective reporting (reporting bias); and 7) other biases. Two investigators conducted the quality assessment for the study methodology, independently and in duplicate. Any divergence was resolved by mutual consensus in the presence of a third investigator.

### Statistical analysis

Statistical analysis of our study was accomplished by an independent statistician using Cochrane systematic review software Review Manager (RevMan; Version 5.3.5., The Nordic Cochrane Centre, The Cochrane Collaboration, Copenhagen, 2014). We used Mann-Whitney *U*-test to verify hypothesis and rendered statistical significance as *z*-value and *P*-value < 0.05, and the results were displayed in Forest plots.

Continuous variables were reported as mean and standard derivation (SD), while dichotomous variables were shown as frequency and proportion. An initial test for clinical, methodological and statistical heterogeneities was conducted, and we used the *χ*^2^ test with *P* < 0.1 and *I*^2^ > 50 % to indicate significance. We also performed the sensitivity analysis to substitute alternative decisions or ranges of values for decisions that were arbitrary or unclear, and tested the publication biases by Funnel plot.

Random-effects model was applied in the presence of statistical heterogeneity; otherwise fixed-effects model was used. For continuous data we calculated MD and 95 % CI, while for dichotomous data we calculated RR and 95 % CI. Furthermore, in terms of pre- and post-bronchodilator FEV_1_, incidence of COPD exacerbation and adverse events, we separately conducted sub-analysis at different follow-up time points.

## Results

**Fig. 1:**
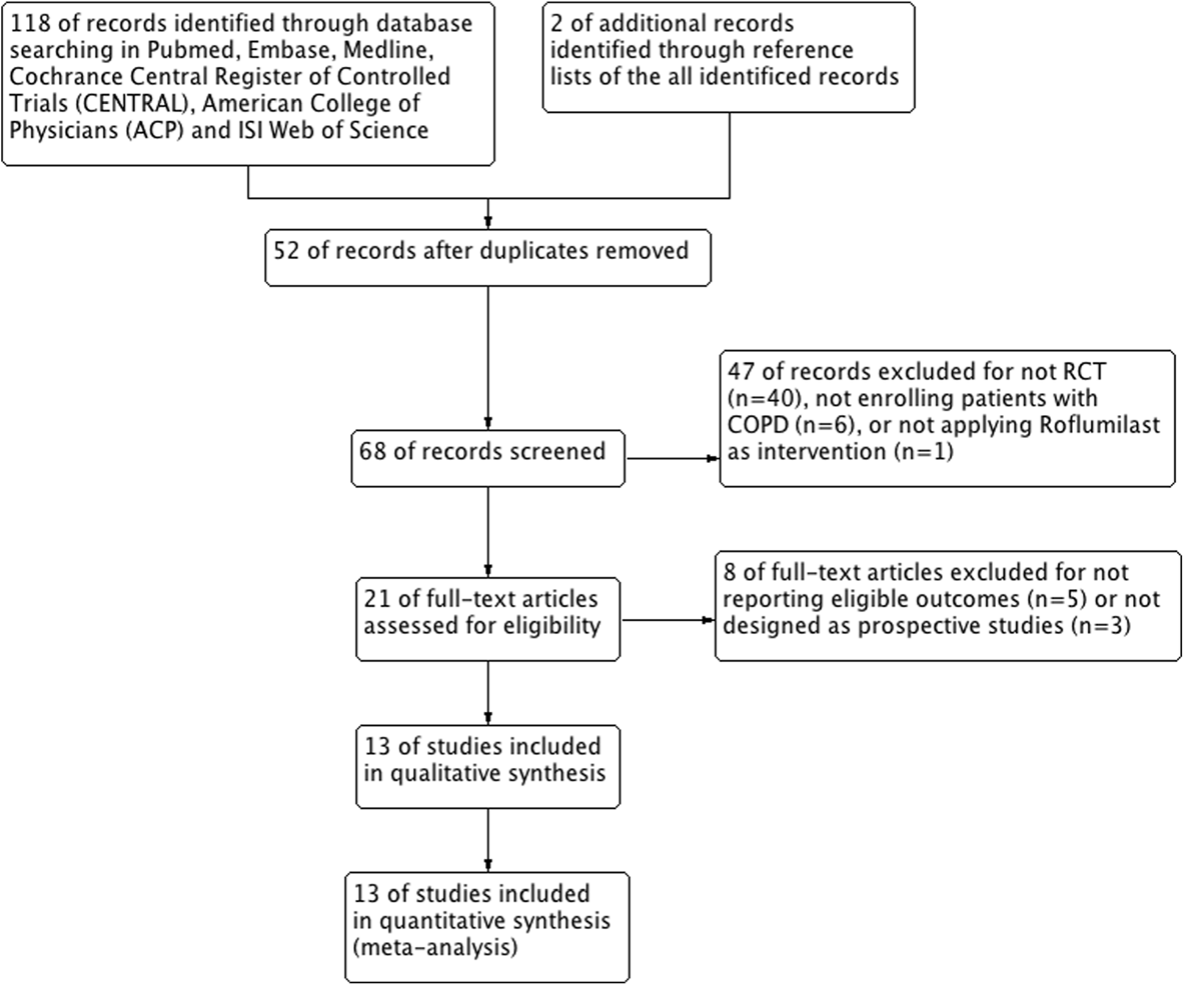
Study flow diagram. ACP, American College of Physician; CENTRAL, Cochrane Central Register of Controlled Trials; COPD, chronic obstructive pulmonary disease; RCT, randomized controlled trial

Initially 120 records were identified, of which 118 were extracted from electronic databases and 2 were extracted from reference lists review. (Fig. 1) By screening the titles and abstracts, 99 studies were discarded for duplication (n = 52), not RCTs (n = 40), patients without COPD (n = 6), and intervention treatment without roflumilast (n = 1). We searched the full-text articles for the remaining 21 studies, and eventually 13 trials [11, 13, 14, 16–25] were enrolled in our final analysis due to 5 studies not reporting eligible outcomes and 3 studies being retrospective studies.

### Study description

All 13 studies enrolled patients with severe COPD, but 2 studies [16, 17] also included mild COPD, 7 studies [11, 13, 14, 16, 17, 20, 23] included moderate COPD, and 6 studies [18, 19, 21, 22, 24, 25] included very severe COPD. Except for one study [13] administering roflumilast plus salmeterol or tiotropium as intervention treatment and placebo plus salmeterol or tiotropium as control, all studies compared roflumilast with placebo. Treatment duration and follow-up period were not identical in different studies with 2 studies [20, 23] for 12 weeks, 1 study [14] for 14 weeks, 4 studies [11, 13, 17, 24] for 24 weeks, 1 study [16] for 26 weeks, and 5 studies [18, 19, 21, 22, 25] for 52 weeks. In terms of the outcome measures, 11 studies [11, 13, 14, 16–21, 23, 24] reported change of pre-bronchodilator FEV_1_, 10 studies [11, 13, 14, 18–21, 23–25] reported change of post-bronchodilator FEV_1_, 8 studies [11, 13, 14, 18–20, 24, 25] provided change of post-bronchodilator FVC, 3 studies [11, 18, 20] provided change of post-bronchodilator FEV_6_, 5 studies [11, 18–20, 23] provided change of post-bronchodilator FEF_25-75_, 4 studies [13, 19, 23, 24] presented data of TDI change, 4 studies [11, 13, 18, 21] presented data of SGRQ change, 10 studies [11, 13, 18–25] showed incidence of COPD exacerbation, and 10 studies [11, 13, 14, 18–21, 23–25] showed incidence of adverse events. Details of patients’ characteristics, intervention strategies, and outcomes were summarized in Tables 1 and 2.

**Table 1:** Details of each enrolled study

Author (Year)		NCT No.	Patients	Population (I/C)	Intervention			Control	Routine	Duration	Follow-up	Outcomes^a^
					Drug	Dose	Frequency					
Bredenbroker, 2002 [16]		FK1 101	Mild to severe COPD	341 (169/172)	Roflumilast	500ug	Once daily	Placebo	Oral	26 weeks	26 weeks	①
Boszormenyi-Nagy, 2005 [17]		FK1 103	Mild to severe COPD	386 (200/186)	Roflumilast	500ug	Once daily	Roflumilast + Placebo	Oral	24 weeks	24 weeks	①
Rabe, 2005 [11]		M2-107	COPD with postbronchodilator FEV_1_% of 30 ~ 80 %, age ≥ 40 years and a smoking history > 10 pack-year	835 (555/280)	Roflumilast	500ug	Once daily	Placebo	Oral	24 weeks	24 weeks	①②③④⑤⑥⑧⑨
Calverley, 2007 [18]		NCT00430729 (M2-112)	COPD with postbronchodilator FEV_1_% of 50 % or less, age ≥ 40 years and a smoking history > 10 pack-year	1513 (760/753)	Roflumilast	500ug	Once daily	Placebo	Oral	52 weeks	52 weeks	①②③④⑤⑥⑧⑨
Calverley, 2009 [19]		NM (M2-124, M2-125)	COPD with postbronchodilator FEV_1_% of 50 % or less, age > 40 years and a smoking history ≥ 20 pack-year	3091 (1537/1554)	Roflumilast	500ug	Once daily	Placebo	Oral	52 weeks	52 weeks	①②③⑤⑦⑧⑨
Fabbri, 2009 [13]	M2-127	NCT00313209	COPD with postbronchodilator FEV_1_% of 40 ~ 70 %, age > 40 years and a smoking history > 10 pack-year	933 (466/467)	Roflumilast + Salmeterol	500ug	Once daily	Salmeterol + Placebo	Oral	24 weeks	24 weeks	①②③⑥⑦⑧⑨
	M2-128	NCT00424268		743 (371/372)	Roflumilast + Tiotropium			Tiotropium + Placebo				①②③⑥⑦⑧⑨
Lee, 2011 [20]		NCT00242320 (M2-119)	COPD with postbronchodilator FEV_1_% of 30 ~ 80 %, age ≥ 40 years and a smoking history > 10 pack-year	410 (203/207)	Roflumilast	500ug	Once daily	Placebo	Oral	12 weeks	12 weeks	①②③④⑤⑧⑨

^a^Outcome measures include: ① Change of prebronchodilator FEV_1_; ② Change of postbronchodilator FEV_1_; ③ Change of postbronchodilator FVC; ④ Change of postbronchodilator FEV_6_; ⑤ Change of postbronchodilator FEF_25-75_; ⑥ Change of SGRQ; ⑦ Change of TDI; ⑧ Incidence of exacerbation; ⑨ Incidence of adverse events

*COPD* chronic obstructive pulmonary disease, *FEF*_*25-75*_ forced expiratory flow between 25 and 75 % of the vital capacity, *FEV*_*1*_ forced expiratory volume in one second, *FEV*_*6*_ forced expiratory volume in six seconds *FVC* forced vital capacity, *I/C* intervention/control, *NM* not mentioned, *No.* numbers, *SGRQ* St George’s Respiratory Questionnaire, *TDI* transition dyspnea index

**Table 2:** Details of each enrolled study

Author (Year)	NCT No.	Patients	Population (I/C)	Intervention			Control	Routine	Duration	Follow-up	Outcomes^a^
				Drug	Dose	Frequency					
Rennard, 2011 [21]	NCT00076089 (M2-111)	COPD with postbronchodilator FEV_1_% of 50 % or less, age ≥ 40 years and a smoking history > 10 pack-year	1173 (567/606)	Roflumilast	500ug	Once daily	Placebo	Oral	52 weeks	52 weeks	①②⑥⑧⑨
Ferguson, 2012 [22]	NCT01443845	Severe-to-very-severe COPD patients with a history of exacerbations	2300 (1150/1150)	Roflumilast	500ug	Once daily	Placebo	Oral	12 months	12 months	⑧
O’Donnell, 2012 [23]	M2-118	COPD with postbronchodilator FEV_1_% of 30 ~ 80 %, age ≥ 40 years and a smoking history ≥ 10 pack-year	250 (127/123)	Roflumilast	500ug	Once daily	Placebo	Oral	12 weeks	12 weeks	①②⑤⑦⑧⑨
Zheng, 2014 [24]	NCT01313494	COPD with postbronchodilator FEV_1_% of 50 % or less, age ≥ 40 years and a smoking history ≥ 10 pack-year	626 (313/313)	Roflumilast	500ug	Once daily	Placebo	Oral	24 weeks	24 weeks	①②③⑦⑧⑨
Martinez, 2015 [25]	NCT01329029 (REACT)	COPD with postbronchodilator FEV_1_% of 50 % or less, age ≥ 40 years and a smoking history ≥ 20 pack-year	1935 (969/966)	Roflumilast	500ug	Once daily	Placebo	Oral	52 weeks	52 weeks	②③⑧⑨
Wells, 2015 [14]	NCT01572948	Moderate-to-severe COPD with age > 40 years and a smoking history > 10 pack-year	27 (11/16)	Roflumilast	500ug	Once daily	Placebo	Oral	30 days	14 weeks (12 weeks for lung function test)	①②③⑨

^a^Outcome measures include: ① Change of prebronchodilator FEV_1_; ② Change of postbronchodilator FEV_1_; ③ Change of postbronchodilator FVC; ④ Change of postbronchodilator FEV_6_; ⑤ Change of postbronchodilator FEF_25-75_; ⑥ Change of SGRQ; ⑦ Change of TDI; ⑧ Incidence of exacerbation; ⑨ Incidence of adverse events

*COPD* chronic obstructive pulmonary disease, *FEF*_*25-75*_ forced expiratory flow between 25 and 75 % of the vital capacity, *FEV*_*1*_ forced expiratory volume in one second, *FEV*_*6*_ forced expiratory volume in six seconds, *FVC* forced vital capacity, *I/C* intervention/control, *NM* not mentioned, *No.* numbers, *SGRQ* St George’s Respiratory Questionnaire, *TDI* transition dyspnea index

A total of 14,563 patients with COPD were pooled from all the included trials in our final meta-analysis, among which 7,398 patients were assigned to receive roflumilast, while 7,165 patients were administered placebo. The majority of patients enrolled in the studies were male (64 ~ 92.6 %), and the mean age of patients ranged from 62 to 68 years old. All patients had a long smoking history, which was estimated to be at least 37 pack-years, and experienced a severe expiratory airflow obstruction with a mean post-bronchodilator predicted FEV_1_ less than 55 %. Details of baseline characteristics of patients in each enrolled study were shown in Table 3.

**Table 3:** Baseline characteristics of patients in each enrolled trial

Author (year)		No.^a^	Age (year, SD)^a^	Sex (Male, %)^a^	BMI (kg/m^2^, SD)^a^	Smoking (Pack-year, SD)^a^	Post-FEV_1_/FVC (%, SD)^a^	Post-FEV_1_ (L, SD)^a^	Post-FEV_1_ (%predicted, SD)^a^	Post-FVC (L, SD)^a^
Bredenbroker, 2002		169	NM	NM	NM	NM	NM	NM	NM	NM
Boszormenyi-Nagy, 2005		200	NM	NM	NM	NM	NM	NM	NM	NM
Rabe, 2005		555	64 (42 ~ 87)	410 (75)	26 (5.0)	41 (20.6)	50 (12)	1.50 (0.48)	54 (13.2)	3.08 (0.85)
Calverley, 2007		760	65 (9.6)	571 (75)	25 (5.0)	42 (22.9)	40.3 (11.2)	1.13 (0.04)	41 (11.0)	NM
Calverley, 2009		1537	64 (9)	1150 (75)	25.8 (5.9)	48 (25)	42.3 (11.2)	1.10 (0.4)	36.1 (10.6)	NM
Fabbri, 2009	M2-127	466	65 (9)	319 (68)	NM	43 (22)	49.8 (9.4)	1.51 (0.4)	54.7 (9.1)	NM
	M2-128	371	64 (9)	262 (71)	NM	43 (22)	52.7 (10.3)	1.55 (0.5)	56.0 (11.6)	NM
Lee, 2011		203	68 (41 ~ 91)	188 (92.6)	22.39 (3.7)	42 (22.1)	50.5 (11.8)	1.41 (0.5)	55.1 (16.5)	NM
Rennard, 2011 (M2-111)		567	64 (8.7)	387 (68.3)	26.0 (5.7)	50 (28.2)	43.3 (10.1)	1.12 (0.4)	36.8 (10.7)	NM
Ferguson, 2012		1150	NM	NM	NM	NM	NM	NM	NM	NM
O’Donnell, 2012		127	60 (9)	93 (73.2)	26.4 (5.0)	41 (20)	51 (11)	NM	56 (12)	NM
Zheng, 2014		313	64.2 (8.76)	283 (90.4)	21.8 (3.42)	37.2 (21.18)	35.78 (9.69)	0.95 (0.35)	36.84 (11.42)	NM
Martinez, 2015		969	65 (8.4)	718 (74)	26.5 (5.47)	48 (24.6)	40.2 (10.81)	1.1 (0.33)	35.4 (9.25)	NM
Wells, 2015		11	62 (7)	7 (64)	NM	47 (26)	53 (12)	NM	45 (12)	NM

^a^Data reported in all patients receiving vitamin D supplementation

*BMI* body mass index, *FEV*_*1*_ forced expiratory volume in one second, *FVC* forced vital capacity, *NM* not mentioned, *No.* numbers, *SD* standard derivation

**Fig. 2:**
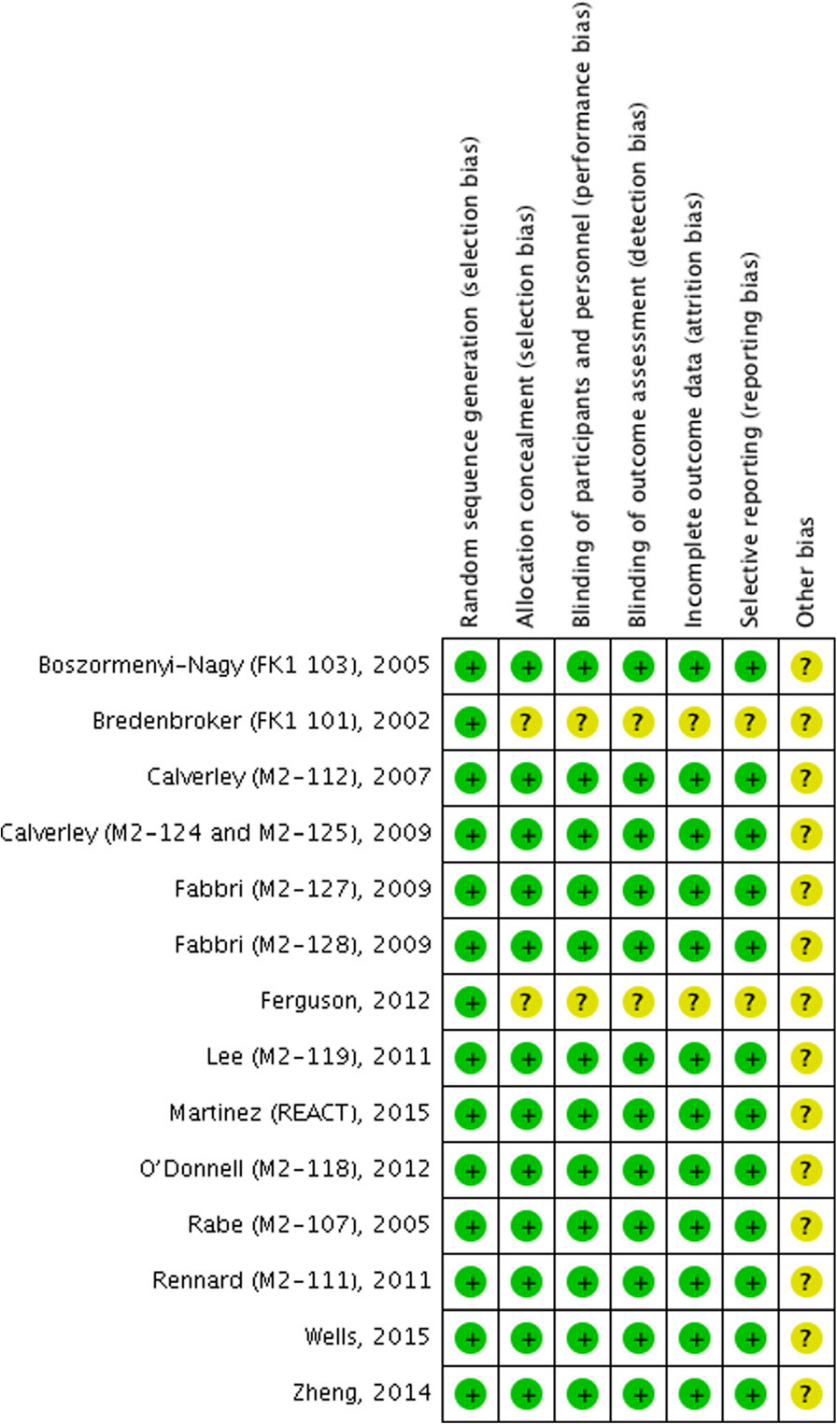
Risk of bias summary

**Fig. 3:**
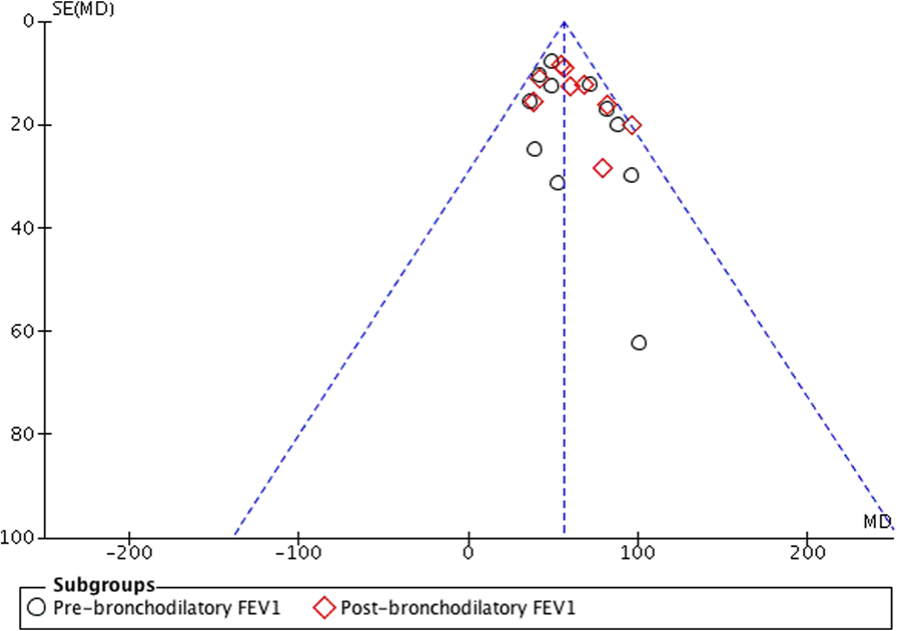
Funnel plot. FEV_1_, forced expiratory volume in one second

Quality assessment of the 13 enrolled studies showed that there was no bias in selection, attribution, or reporting, but 2 studies [16, 22] did not described methods used in allocation concealment and blinding of participants and outcome assessments, neither reported whether the outcome data was incomplete or selective (Fig. 2). No studies excluding for low quality or dubious decisions were found in the sensitivity analysis, and no publication bias was detected in the Funnel plot (Fig. 3).

### Heterogeneity

**Fig. 4:**
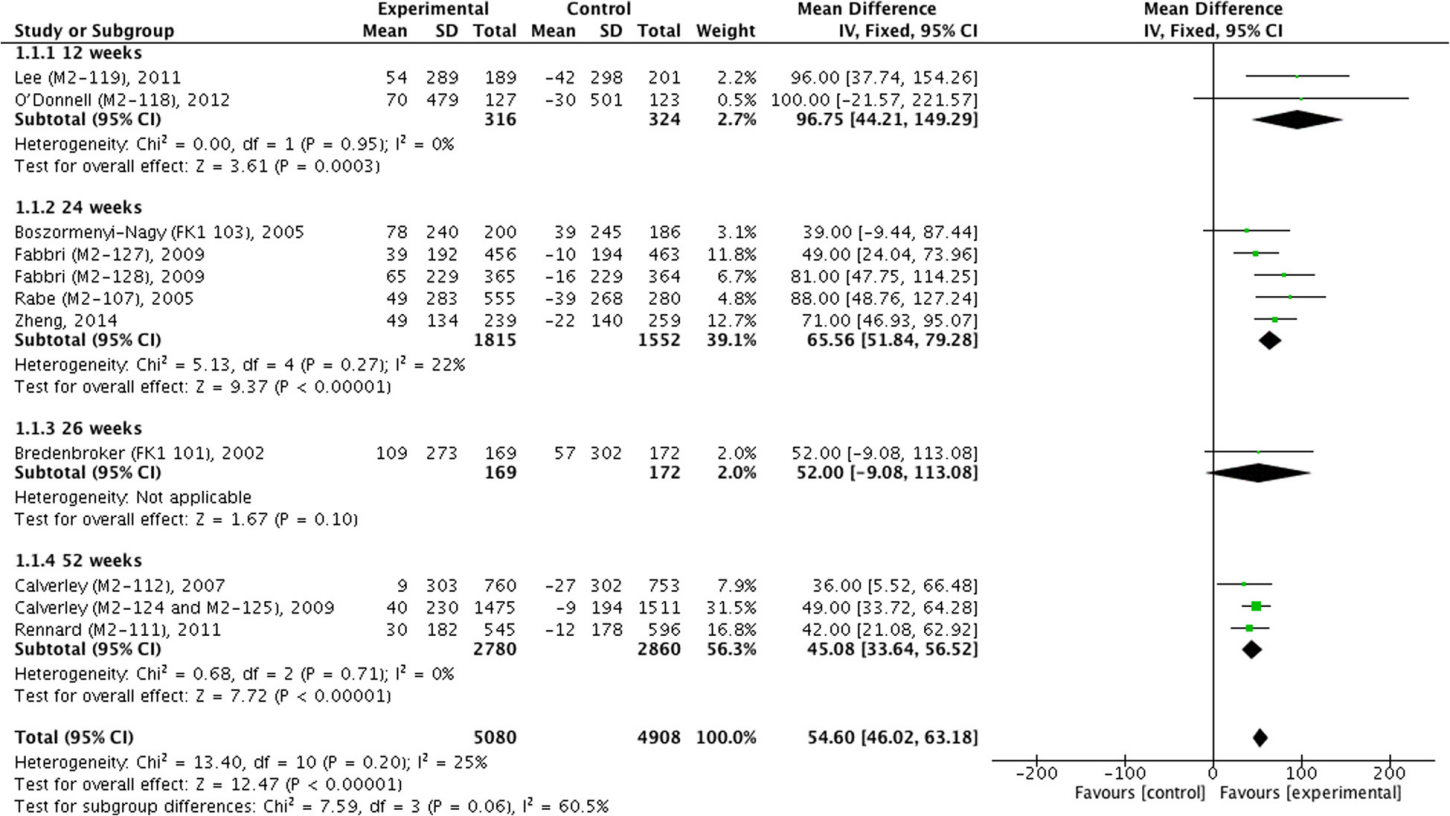
Effects of roflumilast vs. placebo on prebronchodilator FEV_1_. CI, confidence interval; FEV_1_, forced expiratory volume in one second; SD, standard derivation

**Fig. 5:**
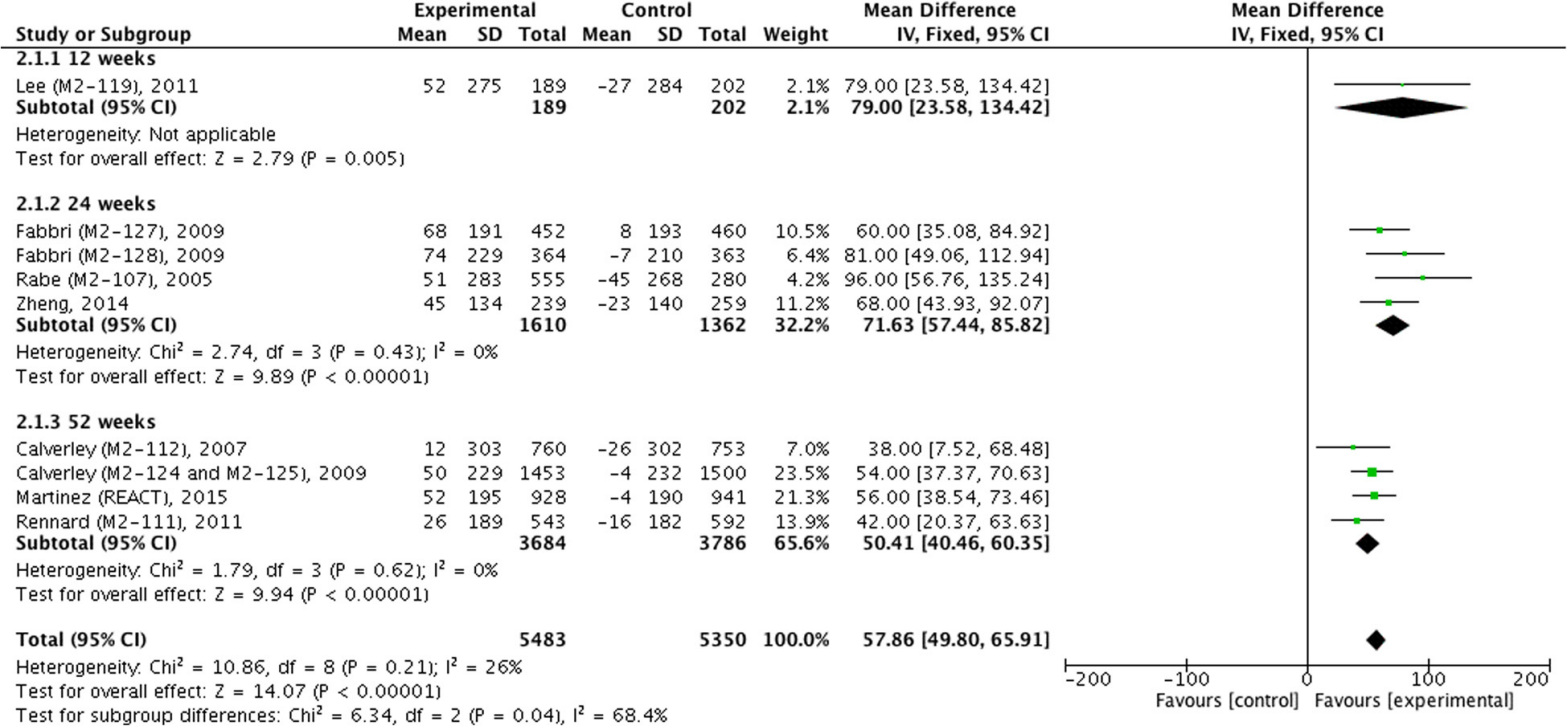
Effects of roflumilast vs. placebo on postbronchodilator FEV_1_. CI, confidence interval; FEV_1_, forced expiratory volume in one second; SD, standard derivation

**Fig. 6:**
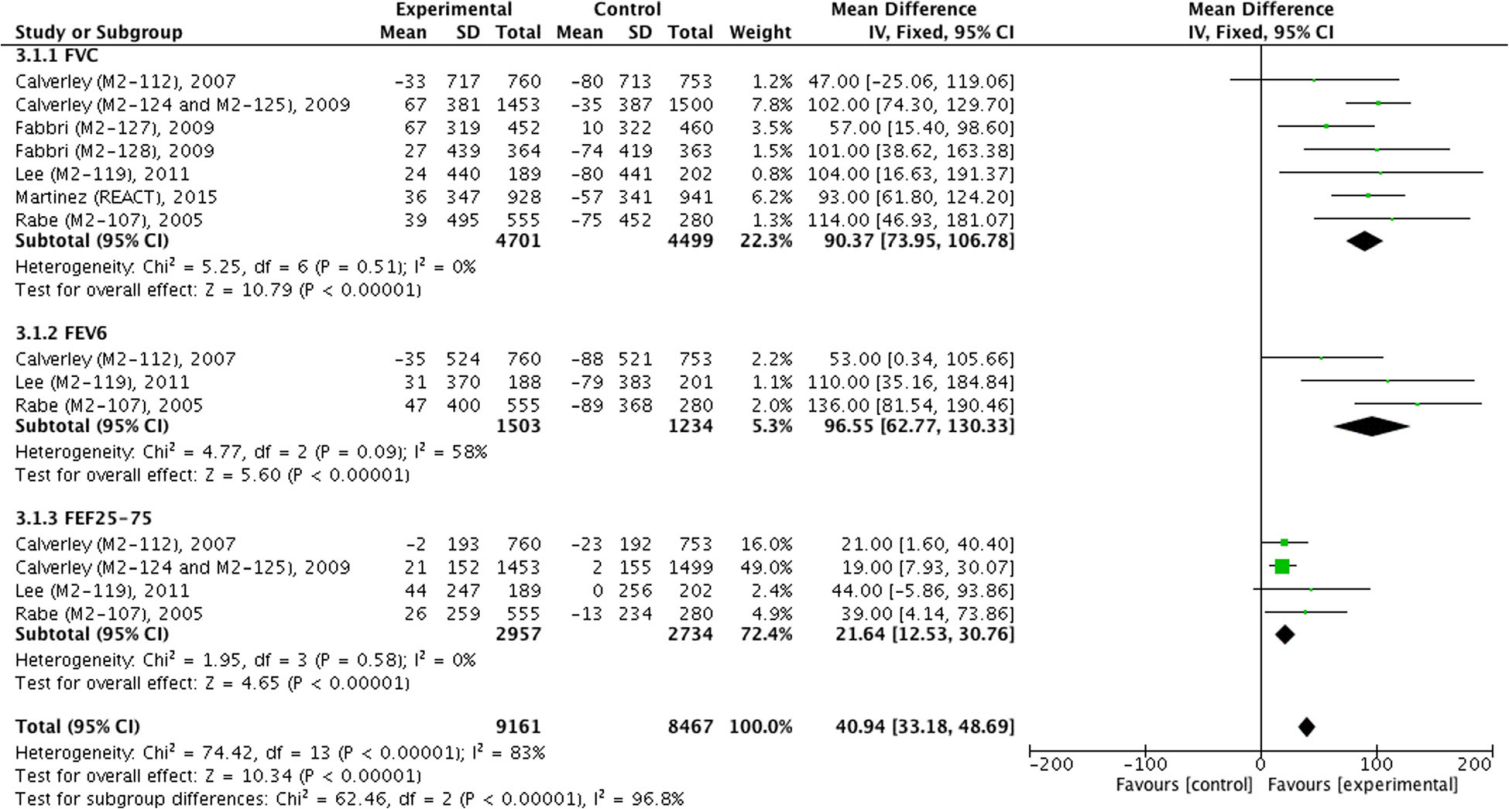
Effects of roflumilast vs. placebo on post-bronchodilator FVC, FEV_6_ and FEF_25-75_. CI, confidence interval; FEF_25-75_, forced expiratory flow between 25 and 75 % of the vital capacity; FEV_6_, force expiratory volume in six seconds; FVC, forced vital capacity; SD, standard derivation

**Fig. 7:**
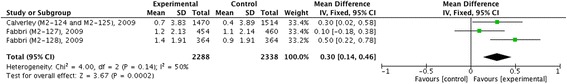
Effect of roflumilast vs. placebo on TDI. CI, confidence interval; SD, standard derivation; TDI, transition dyspnea index

**Fig. 8:**
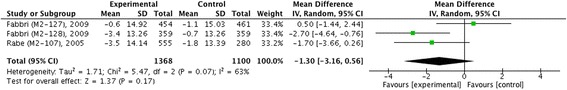
Effect of roflumilast vs. placebo on SGRQ. CI, confidence interval; SD, standard derivation; SGRQ, St George’s Respiratory Questionnaire

**Fig. 9:**
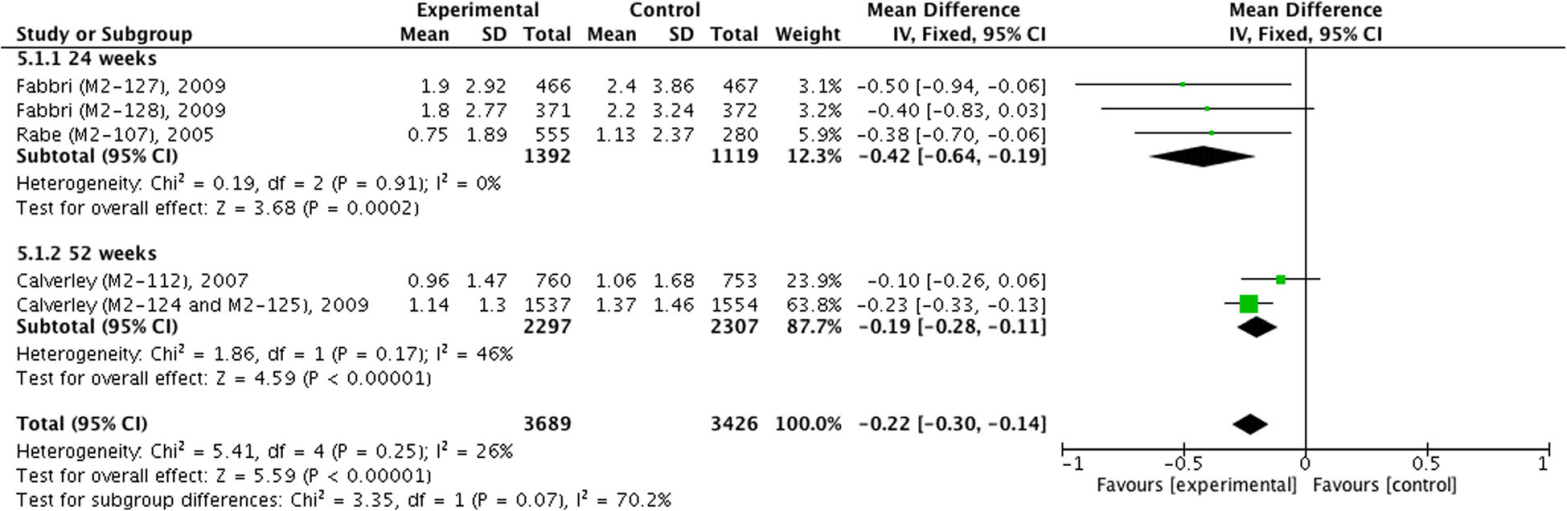
Effect of roflumilast vs. placebo on incidence of COPD exacerbation (number per patient per year). CI, confidence interval; COPD, chronic obstructive pulmonary disease; SD, standard derivation

**Fig. 10:**
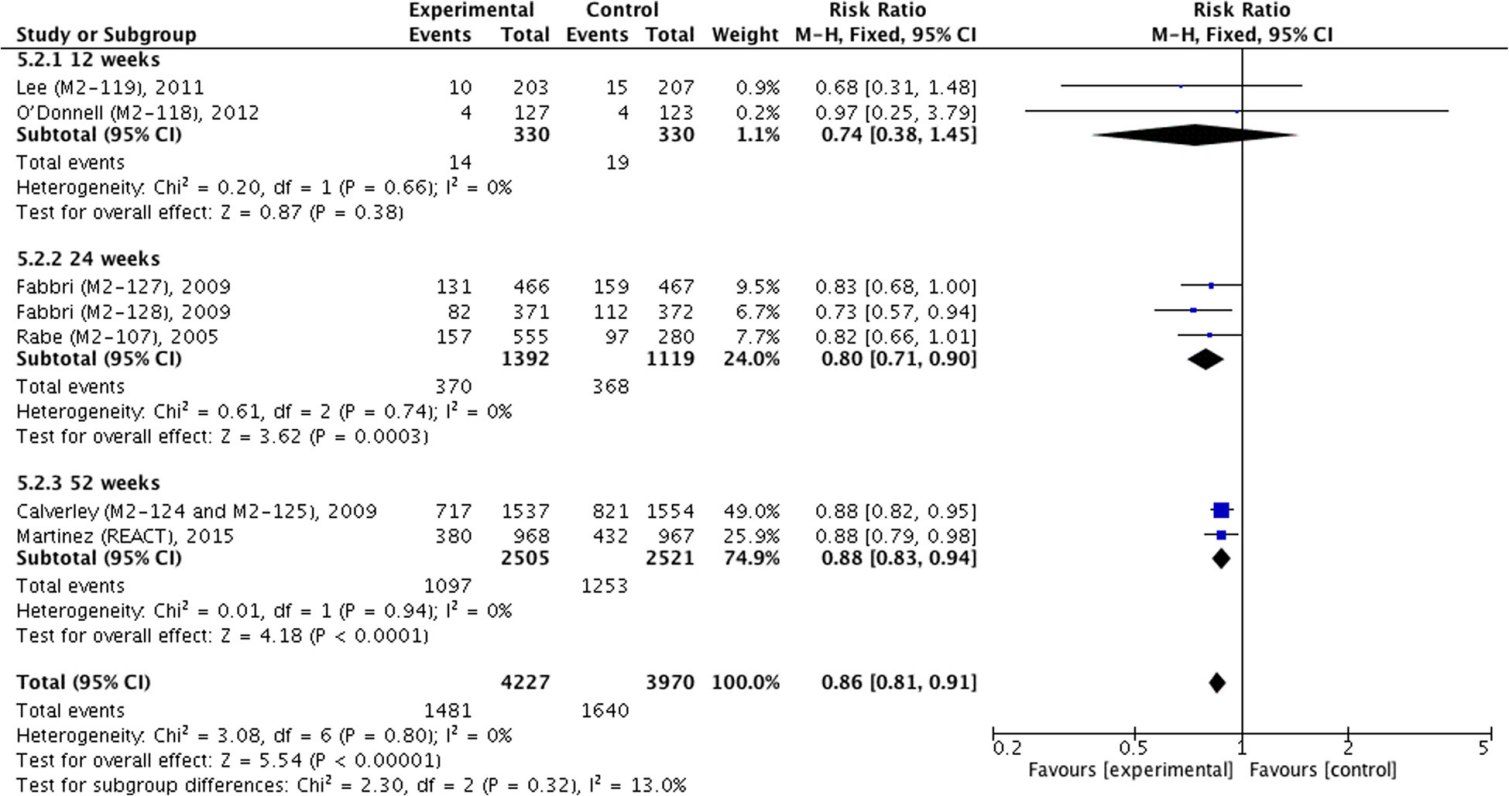
Effect of roflumilast vs. placebo on incidence of COPD exacerbation (patients per total). CI, confidence interval; COPD, chronic obstructive pulmonary disease; M.-H., Mantel-Haenszel

**Fig. 11:**
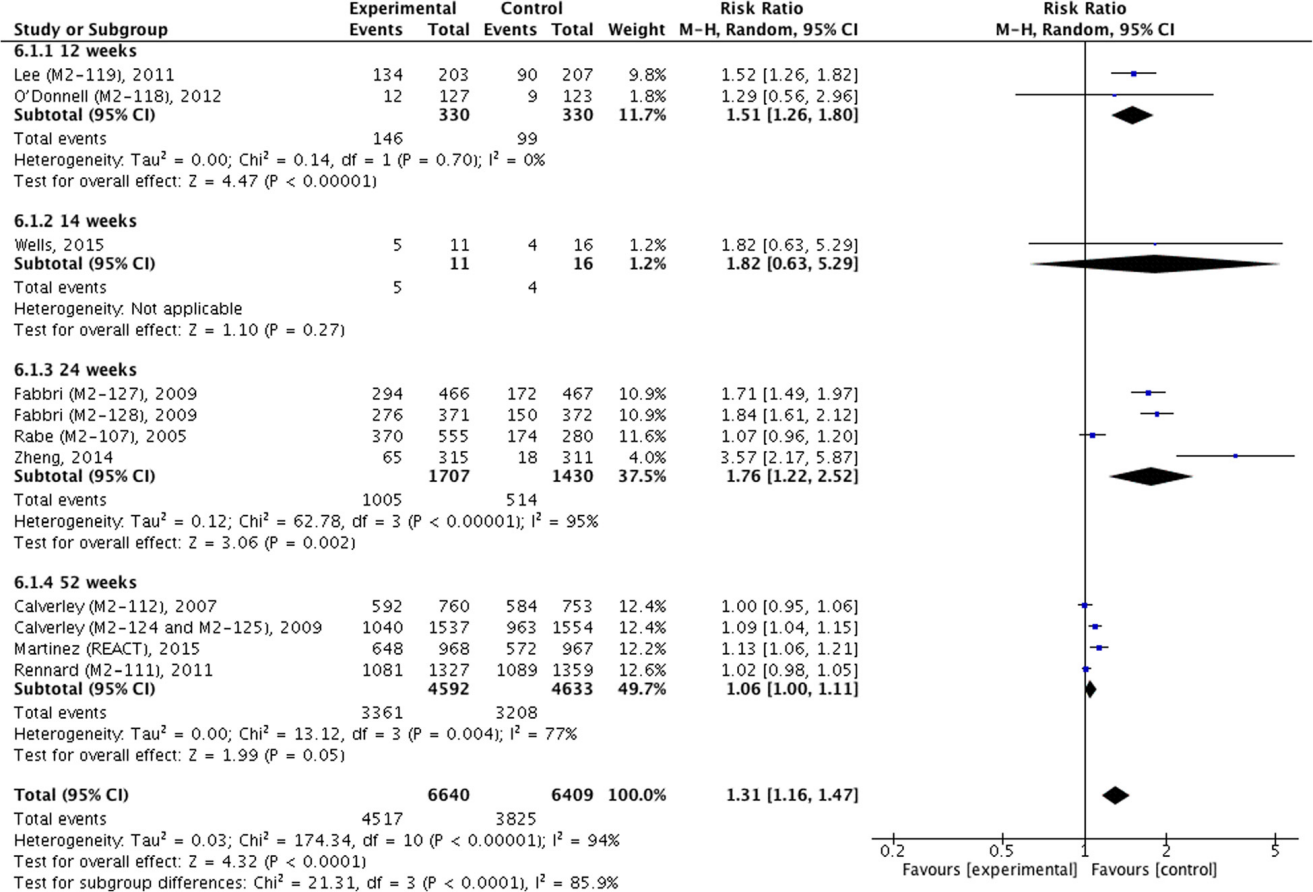
Effect of roflumilast vs. placebo on incidence of adverse events. CI, confidence interval; M.-H., Mantel-Haenszel

We did not find statistical heterogeneity in pre- and post-bronchodilator FEV_1_ (Figs. 4 and 5), postbronchodilaotr FVC and FEF_25-75_ (Fig. 6), TDI (Fig. 7), or incidence of COPD exacerbation (Fig. 9 and 10); whereas significance statistical heterogeneity was found in post-bronchodilator FEV_6_ (*I*^2^ = 58 %, *χ*^2^ = 4.77, *P* = 0.09) (Fig. 6), SGRQ (*I*^2^ = 63 %, *χ*^2^ = 1.71, *P* = 0.07) (Fig. 8), and incidence of adverse events (*I*^2^ = 94 %, *χ*^2^ = 0.03, *P* < 0.001) (Fig. 11).

### Outcomes

#### Lung function

The mean difference of pre-bronchodilator FEV_1_ change from baseline between treatment with roflumilast and placebo in 12 weeks, 24 weeks and 52 weeks were 96.75 ml (95 % CI 44.21 ~ 149.29), 65.56 ml (95 % CI 51.84 ~ 79.28), and 45.08 ml (95 % CI 33.64 ~ 56.52), respectively, which showed that there were significant differences correspondingly (*z* = 3.61, *P* < 0.001; *z* = 9.37, *P* < 0.001; and *z* = 7.72, *P* < 0.001) as well as in overall effects (*z* = 12.47, *P* <0.001) (Fig. 4). As for change of post-bronchodilator FEV_1_ from baseline, we also found significant differences in 24 weeks (MD 71.63, 95 % CI 57.44 ~ 85.82, *z* = 9.89, *P* < 0.001) and 52 weeks (MD 50.41 ml, 95 % CI 40.46 ~ 60.35, *z* = 9.94, *P* < 0.001), and in overall effects (MD 57.86 ml, 95 % CI 49.80 ~ 69.51, *z* = 14.07, *P* < 0.001) (Fig. 5). Meanwhile, significant improvement in change of post-bronchodilator FVC (MD 90.37 ml, 95 % CI 73.95 ~ 106.78, *z* = 10.79, *P* < 0.001), FEV_6_ (MD 96.55 ml, 95 % CI 62.77 ~ 130.33, *z* = 5.60, *P* < 0.001), and FEF_25-75_ (MD 21.64 ml/s, 95 % CI 12.53 ~ 30.76, *z* = 4.65, *P* < 0.001) were found in roflumilast treatment compared with placebo (Fig. 6).

#### Quality of life

Significant improvement of TDI was detected in patients with roflumilast compared with placebo (MD 0.30, 95 % CI 0.14 ~ 0.46, *z* = 3.67, *P* < 0.001) (Fig. 7); whereas, we did not find significant difference in SGRQ between the two treatment groups (MD −1.30, 95 % CI −3.16 ~ 0.56, *z* = 1.37, *P* = 0.17) (Fig. 8).

#### Incidence of COPD exacerbation

Figures 9 and 10 displayed outcomes of incidence of COPD exacerbation in number per patient per year and in proportion, respectively, and both showed that roflumilast significantly decreased COPD exacerbation compared with placebo (MD −0.22, 95 % CI −0.30 ~ −0.14, *z* = 5.59, *P* < 0.001; RR 0.86, 95 % CI 0.81 ~ 0.91, *z* = 5.54, *P* < 0.001). In addition, we also found a significant decrease of COPD exacerbation in patients with roflumilast in 24 weeks (MD −0.42, 95 % CI −0.64 ~ −0.19, *z* = 3.68, *P* < 0.001; RR 0.80, 95 % CI 0.71 ~ 0.90, *z* = 3.62, *P* < 0.001) and 52 weeks (MD −0.19, 95 % CI −0.28 ~ −0.11, *z* = 4.59, *P* < 0.001; RR 0.88, 95 % CI 0.83 ~ 0.94, *z* = 4.18, *P* < 0.001), but no significance was reported in 12 weeks (RR 0.74, 95 % CI 0.38 ~ 1.45, *z* = 0.87, *P* = 0.38).

#### Incidence of adverse events

A great variety of adverse events were detected in patients with roflumilast, among which diarrhea (2.7–12.1 %), weight loss (1.0–12 %), upper respiratory tract infection (2–13.3 %), nasopharyngitis (3.4–8 %), nausea (1.0–6 %), and headache (1.3–4 %) were mainly reported. Discontinuations due to adverse events were more common in patients with roflumilast (5 ~ 14 %) than placebo (2.9 ~ 11 %), however, the incidence of serious adverse events resembled between patients with roflumilast (7 ~ 19 %) and placebo (10 ~ 22 %).

Pooled analysis of 10 studies showed that significantly higher incidence of adverse events were found in patients with roflumilast compared with placebo in 12 weeks (RR 1.51, 95 % CI 1.26 ~ 1.80, z = 4.47, P < 0.001) and 24 weeks (RR 1.76, 95 % CI 1.22 ~ 2.52, z = 3.06, P = 0.002), as well as in overall effects (RR 1.31, 95 % CI 1.16 ~ 1.47, z = 4.32, P < 0.001). However, such a significant difference disappeared in 52 weeks (RR 1.06, 95 % CI 1.00 ~ 1.11, z = 1.99, P = 0.05) (Fig. 11).

## Discussion

In our meta-analysis, we found that roflumilast could significantly improve lung functions, including pre- and post-bronchodilator FEV_1_, FVC, FEV_6_ and FEF_25-75_, alleviate dyspnea symptoms (TDI), and decrease incidence of acute exacerbation, but could not improve quality of life (SGRQ) or decrease early acute exacerbation onset, even with a significant increase of risk of adverse events.

Persistent airflow limitation is the hallmark of COPD, which is also used to evaluate the severity and treatment responses by GOLD guideline [1]. FEV_1_ is the most common spirometric parameter to assess airflow limitation, and the post-bronchodilator FEV_1_ independently divided patients with COPD into 4 stages of severity, and classified them into 4 groups with symptoms and acute exacerbations. Therefore, FEV_1_ improvement is usually rendered as an important factor to identify the treatment efficacy of a new drug for COPD. Previous meta-analyses have reported that roflumilast significantly improved lung function through pre-bronchodilator FEV_1_ compared with placebo, but they did not evaluate the post-bronchodilator FEV_1_ nor consider the onset time of roflumilast in improving FEV_1_ [12, 26, 27]. Our study not only confirmed the effect of roflumilast on improving both pre- and post-bronchodilator FEV_1_, post-bronchodilator FEV_6_ and FEF_25-75_, but also further demonstrated that roflumilast could improve FEV_1_ as early as 12 weeks and the improvement effect lasted for as long as 52 weeks afterward, which we think has important clinical insights because it can facilitate physicians to set up the optimal follow-up plan and determine the administration duration.

It is well known that FVC is a volumetric parameter, which represents lung volume change and is rarely used to assess treatment responses in patients with COPD. Recent years, however, Tashkin and his colleagues conducted a cohort study of 5,756 patients with moderate-to-severe COPD to examine acute bronchodilator responsiveness patterns in theses patients, and they found that mean improvements from baseline were 229 ml in FEV_1_ and 407 ml in FVC, and approximately 49 % of patients with very severe COPD showed a volume response rather than a flow response to the bronchodilators [28], which also revealed the potential value of FVC alteration in evaluating treatment responses in patients with severe airflow limitation and failed to exhibit the requisite threshold increase in FEV_1_. In our study, 6 studies reported the change of FVC from baseline and the final pooled analysis resulted in greater improvement of FVC in patients with roflumilast than placebo. However, we could not compare the mean change of FVC and FEV_1_ or analyze the proportion of patients who responded to FVC and FEV_1_ due to the insufficient data reported. Therefore, future studies focusing on these issues were warranted.

Chronic and progressive dyspnea is one of the typical symptoms in patients with COPD, and is a major cause of disability and impaired quality of life [1]. In our pooled meta-analysis, we found a significant improvement of TDI but without decreasing SGRQ scores in patients with roflumilast compared with placebo. That is, roflumilast could relieve the symptom of dyspnea, but could not attenuate other symptoms including but not limited to cough, sputum, activity endurance, and daily life. As we know, TDI is an evaluative instrument to measure breathlessness related to activities of daily living, and a large of RCTs have demonstrated reliability and accuracy in the characteristics of TDI [29]; while SGRQ is a widely used questionnaire with documented comprehensive measures, and it is recommended that regular treatment for symptoms should be considered if a COPD patient with a symptom score equivalent to SGRQ score ≥ 25 [30–32]. Thus, the different outcomes in the effects of roflumilast on TDI and SGRQ may due to the different content and aspects in each scoring system. However, interpretation of our results should be cautious because of the potential heterogeneity in SGRQ, and the difference of corresponding parts about dyspnea in SGRQ still remains unknown.

COPD exacerbation is an acute event, which can lead to the decline of lung functions and even be fatal to patients [1]. The three meta-analyses mentioned previously also demonstrated the significant decrease of acute exacerbation rate in treatment with roflumilast, but they again did not take the different time points into account when evaluated the effect of roflumilast on affecting incidence of acute exacerbation [12, 26, 27]. Our study illustrated that although roflumilast could significantly reduce the incidence of acute exacerbation, but we did not find such an effect before 24 weeks. Therefore, the improvement of lung function may be earlier than decrease of acute exacerbation, and a minimal treatment duration of 24 weeks might be optimal to achieve improvement of both lung functions and acute exacerbation, which was also explained by a recent RCT with negative effects in FEV_1_ and acute exacerbation due to limited follow-up [14]. Nevertheless, we should notice the limited studies with relatively small samples and the various conditions conducted in different studies with different lengths, and further studies in evaluating acute exacerbation in 12 weeks are needed before a precise conclusion can be drawn.

It has long been recognized that weight loss, malnutrition and skeletal muscle dysfunction are common comorbidities in patients with COPD especially in later stage [33]. Based on the presently available studies, the mostly reported adverse events are diarrhea and weight loss. Our meta-analysis indeed showed a significant increase of adverse event in patients with roflumilast, which further demonstrated the conclusions by Yan and Chong [12, 27], but went contrary to the findings of Oba [26]. However, Rabe and his colleagues also perceived that most adverse events (>90 %) resolved or relieved during the course of the study [11]. Meanwhile, in our study, we also found that the incidence of adverse events between roflumilast and placebo was similar in 52 weeks even though the *P* value was on the borderline. As a result, administration of roflumilast should be cautious with consideration of the treatment benefits and detriments in patients with COPD comorbidities.

Limitations for our meta-analysis are as follows: First, the baseline demographics of patients and extent of airflow limitation in COPD were not identical among the enrolled trials, which may lead to selection biases. Second, potential heterogeneities existed in some outcomes such as post-bronchodilator FEV_6_, SGRQ and incidence of adverse events, which may cause potential confusions to our results and conclusions. Third, the number of studies and patients for pre- and post-FEV_1_ varied in different follow-up groups, especially in 12 weeks, which may also result in inaccurate conclusions. Finally, subgroup analysis of FVC by follow-up time points and comparison of improvement degrees and proportions between FVC and FEV_1_ could not be achieved due to limited data and studies.

## Conclusions

Roflumilast can be considered as an alternative therapy in selective patients with moderate-to-severe COPD due to the effect of lung function improvement, dyspnea alleviation and acute exacerbation decrease but increase of risk of adverse events. More large studies are needed, particularly with different follow-up and treatment duration, to further determine the role of roflumilast, including cost-effectiveness and time-to-survive, in patients with moderate-to-severe COPD.

## Abbreviations

ACP: American College of Physician
cAMP: cyclic adenosine monophosphate
CENTRAL: cochrane central register of controlled trials
CI: confidence interval
COPD: chronic obstructive pulmonary disease
CYP: cytochrome P450
FEF_25-75_: forced expiratory flow between 25 and 75 % of the vital capacity
FEV_1_: forced expiratory volume in one second
FEV_6_: force expiratory volume in six seconds
FVC: forced vital capacity
GBD: global burden of disease
GOLD: global initiative for chronic obstructive lung disease
MD: mean difference
PDE4: phosphodiesterase-4
RCT: randomized controlled trial
RR: risk ratio
SD: standard derivation
SGRQ: St George’s respiratory questionnaire
SOBQ: shortness of breath questionnaire
TDI: transition dyspnea index
